# Establishment and Application of Matrix-Assisted Laser Desorption/Ionization Time-of-Flight Mass Spectrometry for Detection of *Shewanella* Genus

**DOI:** 10.3389/fmicb.2021.625821

**Published:** 2021-02-18

**Authors:** Keyi Yu, Zhenzhou Huang, Ying Li, Qingbo Fu, Lirong Lin, Shiyao Wu, Hang Dai, Hongyan Cai, Yue Xiao, Ruiting Lan, Duochun Wang

**Affiliations:** ^1^National Institute for Communicable Disease Control and Prevention, Chinese Center for Disease Control and Prevention (China CDC), State Key Laboratory for Infectious Disease Prevention and Control, Beijing, China; ^2^Center for Human Pathogenic Culture Collection, China CDC, Beijing, China; ^3^Workstation for Microbial Infectious Disease, Shunyi District Center for Disease Control and Prevention, Beijing, China; ^4^Zybio Inc., Chongqing, China; ^5^School of Biotechnology and Biomolecular Sciences, University of New South Wales, Sydney, NSW, Australia

**Keywords:** MALDI-TOF mass spectrometry, detection, *Shewanella*, multilocus sequence analysis, establishment and application

## Abstract

*Shewanella* species are widely distributed in the aquatic environment and aquatic organisms. They are opportunistic human pathogens with increasing clinical infections reported in recent years. However, there is a lack of a rapid and accurate method to identify *Shewanella* species. We evaluated here matrix-assisted laser desorption/ionization time-of-flight mass spectrometry (MALDI-TOF MS) for rapid identification of *Shewanella.* A peptide mass reference spectra (PMRS) database was constructed for the type strains of 36 *Shewanella* species. The main spectrum projection (MSP) cluster dendrogram showed that the type strains of *Shewanella* species can be effectively distinguished according to the different MS fingerprinting. The PMRS database was validated using 125 *Shewanella* test strains isolated from various sources and periods; 92.8% (*n* = 116) of the strains were correctly identified at the species level, compared with the results of multilocus sequence analysis (MLSA), which was previously shown to be a method for identifying *Shewanella* at the species level. The misidentified strains (*n* = 9) by MALDI-TOF MS involved five species of two groups, i.e., *Shewanella algae*–*Shewanella chilikensis*–*Shewanella indica* and *Shewanella seohaensis*–*Shewanella xiamenensis*. We then identified and defined species-specific biomarker peaks of the 36 species using the type strains and validated these selected biomarkers using 125 test strains. Our study demonstrated that MALDI-TOF MS was a reliable and powerful tool for the rapid identification of *Shewanella* strains at the species level.

## Introduction

The genus *Shewanella* comprises a group of oxidase-, catalase-, and ornithine decarboxylase-positive and H_2_S-producing, facultative anaerobic bacteria with a wide distribution in the environment. It plays an important ecological role in many fields such as materials engineering, environmental engineering (Zou et al., 2018), and marine biology (Gorby et al., 2006; Fredrickson et al., 2008; Kouzuma et al., 2015; Daeffler et al., 2017). However, *Shewanella* is a common source of food spoilage bacteria, in particular seafood; and *Shewanella* contamination of foods during food processing and storage adversely affects the production, transportation, and sales (Hau and Gralnick, 2007; McLean et al., 2008; Wang et al., 2009; Janda and Abbott, 2014). *Shewanella* is also an opportunistic pathogen of humans (Erfanmanesh et al., 2019). Through occupational or recreational activities, exposure to the marine environment containing *Shewanella* or ingestion of marine organisms contaminated by *Shewanella* (Janda and Abbott, 2014) may cause a range of infections including skin and soft tissue infections (SSTIs), invasive diseases, hepatobiliary diseases, otitis media and associated sequelae, and other infections (Janda and Abbott, 2014). Some studies have shown that SSTI is the most common clinical features of *Shewanella* infection, including cellulitis, abscess, or necrotizing fasciitis (Yousfi et al., 2017). In recent years, *Shewanella* has been isolated from more and more clinical specimens (Janda and Abbott, 2014).

The genus of *Shewanella* has a high diversity with more than 70 species reported so far^1^. An efficient and accurate method for the identification of *Shewanella* species is still lacking. Traditional methods are mainly based on phenotypic tests combined with biochemical identification, which is time-consuming, laborious, and even inaccurate. The widely used 16S rRNA gene as bacterial species identification tool (Yarza et al., 2014) has been found to lack the power to identify *Shewanella* at the species level (Sun et al., 2013; Glaeser and Kämpfer, 2015). The housekeeping gene *gyrB* was found to have a higher resolution than 16S rRNA for *Shewanella* species identification (Bozal et al., 2002; Miyazaki et al., 2006; Sung et al., 2012), but no standardized cutoff value has been established for the identification (Bozal et al., 2002; Miyazaki et al., 2006; Sung et al., 2012). We previously established that the method of multilocus sequence analysis (MLSA) can accurately identify *Shewanella* at the species level (Fang et al., 2019). This method requires PCR and sequencing of six housekeeping genes from each isolate, which is time-consuming and costly. Thus, the method is unsuitable for clinical diagnostic laboratories.

Matrix-assisted laser desorption/ionization time-of-flight mass spectrometry (MALDI-TOF MS) has become a powerful technology for rapid microbial identification in recent years. It has a short turnaround time for species identification to enable targeted treatment. The principle of MALDI-TOF MS based on species identification is that each species has its characteristic MS spectra that provide a good resolution for differentiation of bacteria at the species level (van Belkum et al., 2015). MALDI-TOF MS has been rapidly developed and widely used in clinical laboratories (Angeletti, 2017; Schubert and Kostrzewa, 2017), due to its advantages of convenient sample preparation, simple experimental operation, and high identification accuracy (Samantha et al., 2018). A key requirement of MALDI-TOF MS based on species identification is a specific and accurate spectra database (Jang and Kim, 2018), which must be established for the targeted species.

The objectives of this study were to establish a peptide mass reference spectra (PMRS) database of *Shewanella* species by MALDI-TOF MS and to validate the effectiveness of MALDI-TOF MS and the database for identifying *Shewanella* species, using type strains of 36 species and 125 test strains derived from clinical, environmental, and food samples.

## Materials and Methods

### Bacterial Strains and Cultivation Methods

A total of 161 *Shewanella* strains were used in this study including 36 type strains and 125 test strains. All type strains were sourced from the China General Microbiological Culture Collection Center (CGMCC), the German Collection of Microorganisms and Cell Cultures [Deutsche Sammlung von Mikroorganismen und Zellkulturen (DSMZ)], the Japan Collection of Microorganisms (JCM), the Korean Collection for Type Cultures (KCTC), the Belgian Co-ordinated Collections of Micro-organisms (BCCM/LMG Bacteria Collection), and the Marine Culture Collection of China (MCCC). Detailed information of type strains is listed in Table 1. The three non-*Shewanella* strains, namely, *Vibrio cholerae* N16961, *Pseudomonas aeruginosa* 09MAS0023, and *Aeromonas hydrophila* 2247, were sourced from Center for Human Pathogenic Culture Collection (CHPC). The test strains were isolated from different sources in China, during years 2007–2020, including clinical (*n* = 75), food (*n* = 39), and environmental (*n* = 11) isolates. The 36 *Shewanella* type strains were incubated on marine agar 2216E at suitable temperatures according to the protocols provided by each pathogen culture collection center. The test strains were identified by API20E (bioMérieux SA) according to instrument of manufactory and incubated at 37°C for 18–24 h for subsequent identifications. *Escherichia coli* ATCC 25922 was used for the calibration of the instrument.

**TABLE 1:** Detailed information of 36 *Shewanella* type strains.

No.	Species	Strain	Isolation place	Isolation source	Isolation year#
1	*Shewanella aestuarii*	JCM 17801^T^	Suncheon Bay, Korea	A tidal flat	2011
2	*Shewanella algae*	JCM 21037^T^	Japan	Red alga	1990
3	*Shewanella algicola*	KCTC 23253^T^	Jeju Island, Korea	Brown alga, *Sargassum thunbergii*	−
4	*Shewanella algidipiscicola*	LMG 23746^T^	Baltic Sea, Denmark	Plaice	2001
5	*Shewanella aquimarina*	JCM 12193^T^	Yellow Sea, Korea	Seawater	−
6	*Shewanella baltica*	DSM 9439^T^	Japan	Oil brine	1998
7	*Shewanella basaltis*	KCTC 22121^T^	Jeju Island, Korea	Marine black sand	−
8	*Shewanella carassii*	08MAS2251^T^	Anhui, China	Surface of crucian carp, *Carassius carassius*	2008
9	*Shewanella chilikensis*	KCTC 22540^T^	Orissa, India	Sediment of a lagoon	2007
10	*Shewanella corallii*	DSM 21332^T^	Red Sea, Israel	A coral	2005
11	*Shewanella decolorationis*	JCM 21555^T^	Guangzhou, China	Activated sludge	2002
12	*Shewanella dokdonensis*	KCTC 22898^T^	East Sea, Korea	Seawater	2006
13	*Shewanella electrodiphila*	DSM 24955^T^	Mid-Atlantic Ridge	Deep-sea sediment	2007
14	*Shewanella gaetbuli*	KCTC 22431^T^	Korea	A tidal flat	−
15	*Shewanella gelidii*	MCCC 1K00697^T^	Yellow Sea, China	Red alga, *Gelidium amansii*	2014
16	*Shewanella glacialipiscicola*	LMG 23744^T^	Baltic Sea, Denmark	Cod	1996
17	*Shewanella hafniensis*	KCTC 22180^T^	Baltic Sea, Denmark	Cod	2001
18	*Shewanella hanedai*	DSM 6066^T^	Arctic Ocean	Marine sediment	−
19	*Shewanella indica*	KCTC23171^T^	Karwar jetty, India	Sediment of the Arabian Sea	2006
20	*Shewanella inventionis*	KCTC 42807^T^	Okinawa Trough	Deep-sea sediment	2014
21	*Shewanella kaireitica*	DSM 17170^T^	Suruga Bay, Japan	Deep-sea sediment	−
22	*Shewanella litorisediminis*	KCTC 23961^T^	Saemankum, Korea	A tidal flat sediment	−
23	*Shewanella livingstonensis*	LMG 19866^T^	Johnson’s Dock, Antarctica	Water	−
24	*Shewanella mangrovi*	MCCC 1A00830^T^	Fujian, China	Mangrove sediment	2013
25	*Shewanella marinintestina*	JCM 11558^T^	Yokohama, Japan	Squid body	1994
26	*Shewanella marisflavi*	JCM 12192^T^	Yellow Sea, Korea	Seawater	−
27	*Shewanella olleyana*	LMG 21437^T^	Tasmania, Australia	Saline waters of estuary	1998
28	*Shewanella pacifica*	KCTC 12235^T^	Chazhma Bay, Japan	Seawater	−
29	*Shewanella pneumatophori*	KCTC 23973^T^	Japan	The intestines of Pacific mackerel, *Pneumatophorus japonicus*	1987
30	*Shewanella profunda*	JCM 12080^T^	Pacific Ocean	Deep marine sediment	2000
31	*Shewanella putrefaciens*	ATCC 8071^T^	England	Butter	1931
32	*Shewanella sairae*	MCCC 1A01705^T^	Pacific Ocean	Saury intestine	1995
33	*Shewanella schlegeliana*	JCM 11561^T^	Hiroshima, Japan	Black porgy intestine	1998
34	*Shewanella seohaensis*	KCTC 23556^T^	Saemankum, Korea	A tidal flat sediment	−
35	*Shewanella vesiculosa*	LMG 24424^T^	Deception Island	Marine sediments	−
36	*Shewanella xiamenensis*	MCCC 1A00763^T^	Fujian, China	Coastal sea sediment	−

*# “−” means isolation year unknown.*

### Sample Preparation for Matrix-Assisted Laser Desorption/Ionization Time-of-Flight MS

An ethanol/formic acid method was used for protein extraction (Anja and Sascha, 2009). One loop of fresh bacterial culture was thoroughly suspended in 300 μl of ultrapure water, and then 900 μl of absolute ethanol was added. The mixtures were centrifuged at 14,000 × *g* for 5 min. Then the supernatant was discarded, and the pellet was allowed to dry at room temperature. Next, equal volumes of 70% formic acid and acetonitrile were added to the pellet in sequence. After the pellet was well suspended, it was centrifuged at 14,000 × *g* for 3 min, and the whole cell extracts were transferred to a clean tube. The matrix solution consisted of acetonitrile (500 μl), ultrapure water (475 μl), trifluoroacetic acid (25 μl), and supersaturated α-cyano-4-hydroxycinnamic acid (CHCA) (Zybio Inc., Chongqing, China). One microliter of the protein extracts was spotted onto a 96-well target plate (Zybio Inc., Chongqing, China). Each spot was overlaid with 1 μl of matrix solution and allowed to dry at room temperature.

### Parameter Setting, Spectrum Generation, and Identification

Mass spectrometry analysis was performed using MALDI-TOF MS EXS3000 (Zybio Inc., Chongqing, China). MS spectra were obtained in linear mode within a range of 2,000–20,000 Da. *E. coli* ATCC 25922 was used for mass calibration and instrument parameter optimization, to make the average deviation of molecular weight less than 300 ppm after correction. MS data were analyzed by MDT Master (version 1.1). As specified by the manufacturer’s instructions, log scores ≥2.0 were accepted for the identification at the species level, and log scores <2.0 and ≥1.7 were taken as the identification at the genus level or the presumptive species level identification. Log scores below 1.7 were considered unreliable.

Each sample was coated with 12 targets. At least 24 high-quality spectra with stable baseline, abundant protein peaks and even distribution were selected. The software of iDBac (version 1.1.10) was used to create the dendrogram based on the main spectrum projection (MSP), using the algorithm of unweighted pair-group method with arithmetic means (UPGMA). Three non-*Shewanella* strains were included as controls.

### Multilocus Sequence Analysis of *Shewanella* Test Strains

Genomic DNAs were extracted according to the standardized instructions of the DNA extraction kit (TaKaRa, Dalian, China). Six single-copy housekeeping genes (*gyrA*, *gyrB*, *infB*, *recN*, *rpoA*, and *topA*) were selected according to previous studies (Fang et al., 2019). Housekeeping genes of the 36 type strains were obtained from GenBank (Supplementary Table 1). DNAStar software was used to join the upstream and downstream sequences. MEGA 6.06 was used to compare the concatenated sequences and build the neighbor-joining phylogenetic tree. In terms of parameter setting of the evolutionary tree, Kimura’s two-parameter model with the pairwise-deletion option was used. The robustness of tree topologies was evaluated with 1,000 bootstrap replications, and values >70% were shown at the nodes of the branches.

### Analysis of Specific Biomarker Peaks in Mass Spectrometry

Mass spectrometry analysis was performed using the MDT Master software to calculate the height and area of spectrum peaks, and Welch’s *t* test was used to determine peaks with statistical differences. Finally, an output file was generated. The peaks with relative signal intensity greater than 2% were extracted, and normality test (*W* test) was performed on the distribution of the peaks. Next, Kruskal–Wallis *H* test (when *P-*value < 0.1 in the *W* test) was used to test pairwise difference of the peak distribution between strains. In all cases, *P-*value < 0.05 was considered significant. The spectra peaks from different type strains were collected together, and non-redundant candidate species-specific biomarker peaks were computed for the 36 *Shewanella* species using MDT Master software.

## Results

### Construction of Matrix-Assisted Laser Desorption/Ionization Time-of-Flight MS Database for *Shewanella* Genus

**FIGURE 1:**
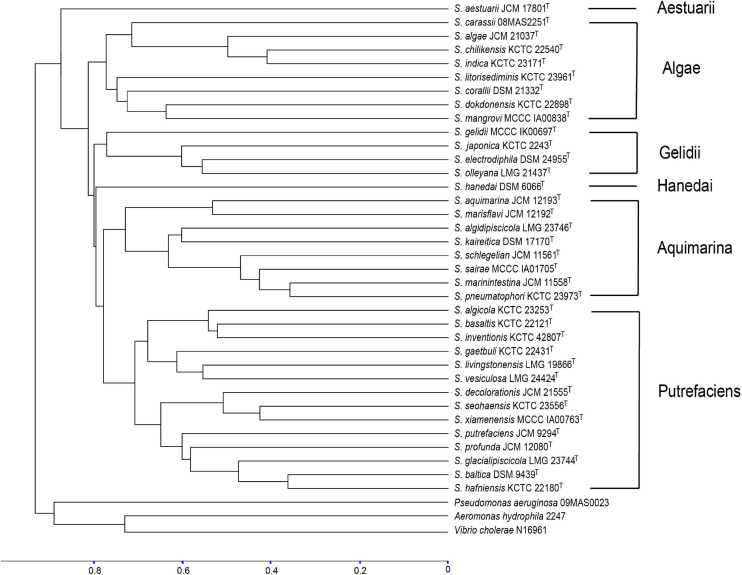
Dendrogram of the cluster analysis of matrix-assisted laser desorption/ionization time-of-flight (MALDI-TOF) mass spectra. The scale below the dendrogram represents the degree of difference in the mass spectrometry fingerprinting of the 36 *Shewanella* type strains, and the difference level reflects the relationship between each other, with the value between 0 and 1. With the main spectrum projection (MSP) similarity of 77.6% as the critical value, 36 type strains of *Shewanella* were divided into six groups. Among them, Algae, Gelidii, Aquimarina, and Putrefaciens included multiple type strains (species); *S. aestuarii* and *S. hanedai* stood separately in their own clade.

In this study, the PMRS database containing 36 type strains of different *Shewanella* species was constructed. About 100 peaks were detected in the MS fingerprinting of each strain, where a large majority of the peaks were concentrated in the range of 3,000–10,000 *m*/*z*. The dendrogram based on MSP of 36 *Shewanella* showed long terminal branches that separate species from the nearest counterparts (Figure 1), and the variance of peaks distribution between branches ranged from 33.9% (*S. marinintestina*–*S. pneumatophore*) to 84.5% (*S. aestuarii*). As the only genus in the family of *Shewanellaceae*, there was large difference in spectra between *Shewanella* and its closely related genera, which were considered as outgroups. No misidentification was observed at the genus level. The 36 type strains of different *Shewanella* species were divided into six clades, using the 77.6% difference in MSP as the cutoff value. The clade of Algae, Gelidii, Aquimarina, and Putrefaciens included multiple species, while *S. aestuarii* and *S. hanedai* were well separated as single species clades from the other clades.

### Species Identification of Test Strains by Multilocus Sequence Analysis

**FIGURE 2:**
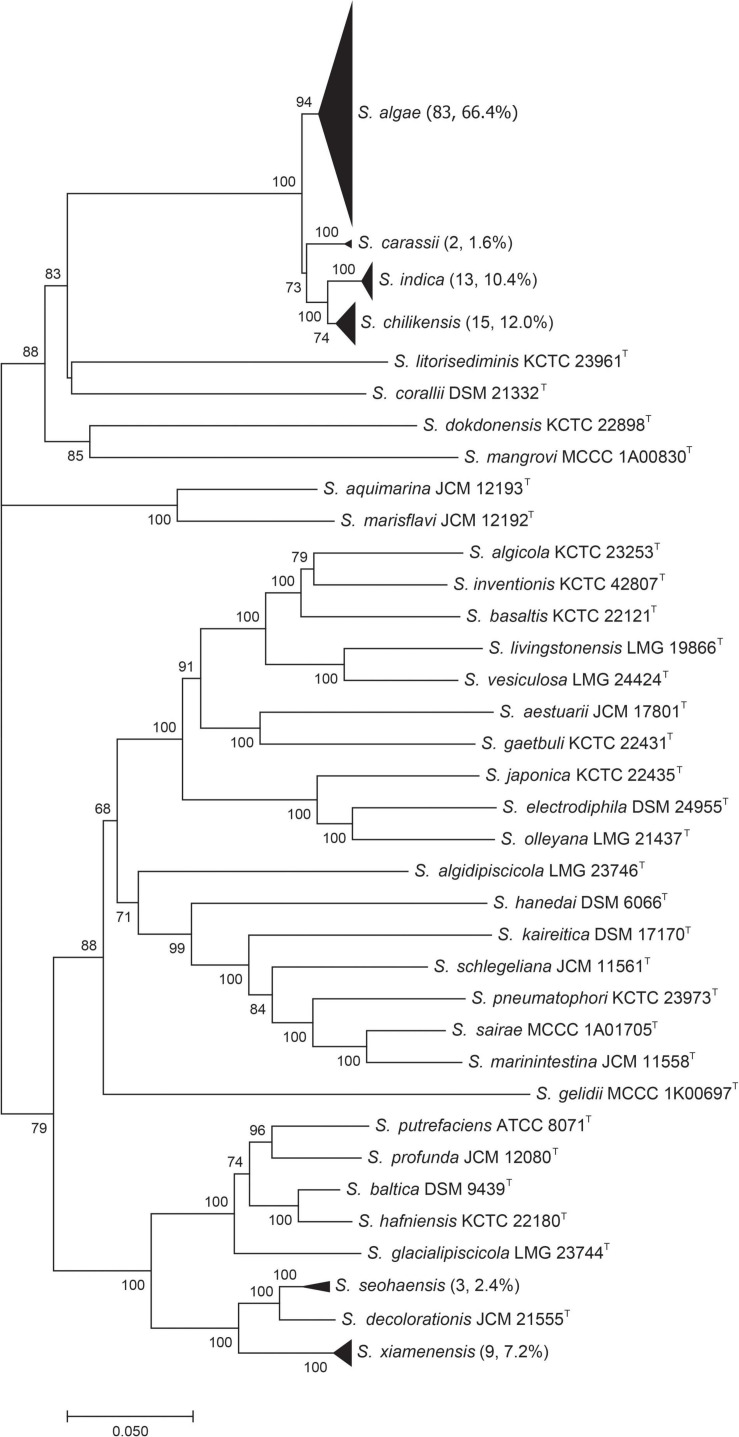
Phylogenetic tree constructed based on six concatenated gene sequences [*gyrA*, *gyrB*, *infB*, *recN*, *rpoA*, and *topA* (4,191 bp)] of 36 *Shewanella* type strains and 125 test strains (by the neighbor-joining method). Each black triangle contains the test strains of the same species, and the number of test strains is shown in brackets. Numbers on branches are bootstrap values in percentage from 1000 replicates.

Multilocus sequence analysis was used to provide a reference identification of all test strains. The concatenated sequences data were used to construct a phylogenetic tree (Figure 2). The test strains were clustered with type strains for unambiguous species identification. Among the 125 strains, 83 (66.4%) were *S. algae*, 15 (12.0%) *S. chilikensis*, 13 (10.4%) *S. indica*, 9 (7.2%) *S. xiamenensis*, 3 (2.4%) *S. seohaensis*, and 2 (1.6%) *S. carassii*. Except for *S. seohaensis* with all isolates from environmental specimens, the other five species contained isolates from clinical samples.

### Species Identification of Test Strains by Matrix-Assisted Laser Desorption/Ionization Time-of-Flight MS

Under the condition of signal-to-noise ratio >3.0, approximately 100 peaks were detected in the range of 2,000 – 20,000 *m*/*z*. When identifying the test strains, online comparison searches were performed against an expanded database (Zybio Inc., Chongqing, China, containing the *Shewanella* PMRS database). The final MALDI-TOF MS identification results for the 125 test strains were *S. algae* (*n* = 87), followed by *S. chilikensis* (*n* = 14), *S. indica* (*n* = 10), *S. xiamenensis* (*n* = 8), *S. seohaensis* (*n* = 4), and *S. carassii* (*n* = 2).

### Comparison of Results Between Multilocus Sequence Analysis and Matrix-Assisted Laser Desorption/Ionization Time-of-Flight MS

**FIGURE 3:**
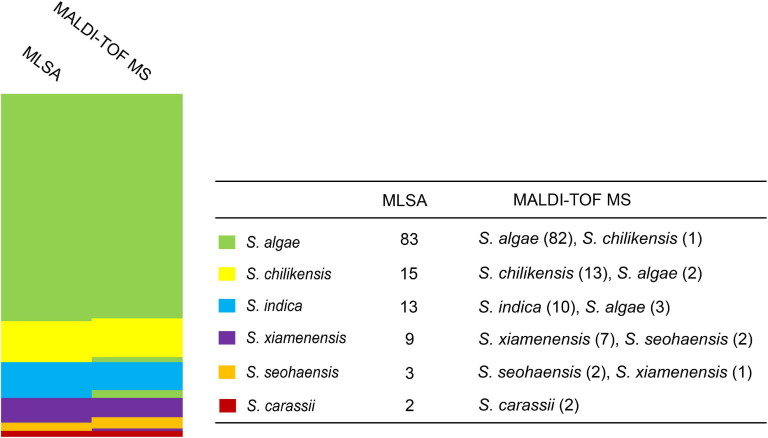
Comparison of identification between multilocus sequence analysis (MLSA) and matrix-assisted laser desorption/ionization time-of-flight mass spectrometry (MALDI-TOF MS) for 125 test strains. Different colors represent different *Shewanella* species.

Since MLSA has been confirmed to be accurate for identifying *Shewanella* at the species level (Fang et al., 2019), all 125 test strains were analyzed by MLSA, and the results were used to evaluate the effectiveness of MALDI-TOF MS for species identification. Taking the MLSA identification results as the “true species identity” of a test strain, all strains were identified correctly at the genus level, and 116 (92.8%) of the test strains were accurately identified at the species level. Nine strains were misidentified by MALDI-TOF MS, involving five species of two groups, i.e., *S. algae*–*S. chilikensis–S. indica* and *S. seohaensis*–*S. xiamenensis*. More specifically, one *S. algae* strain was misidentified as *S. chilikensis*, while the two *S. chilikensis* strains and the three *S. indica* strains were wrongly identified as *S. algae*; two strains of *S. xiamenensis* were misidentified as *S. seohaensis*, and one strain of *S. seohaensis* was wrongly identified as *S. xiamenensis* (Figure 3).

### Analysis of Potential Species-Specific Biomarker Peaks

**FIGURE 4:**
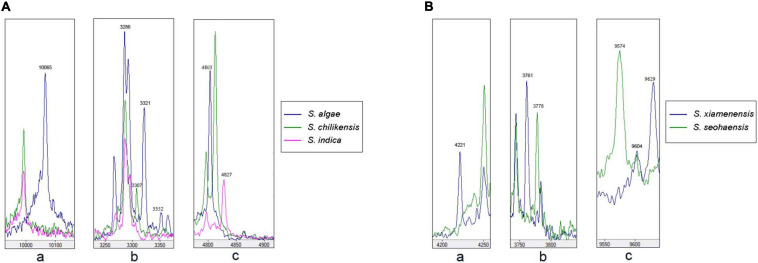
Matrix-assisted laser desorption/ionization time-of-flight mass spectrometry (MALDI-TOF MS) spectra of the five species displayed in the MDT Master: **(A)** **(a)** Peak at 10,065 *m*/*z* of *S. algae* compared with *S. chilikensis* and *S. indica*; **(b)** comparison of peak at 3,307 *m*/*z* belonging to *S. chilikensis* to *S. algae*, *S. indica*, respectively. **(c)** Different distribution of peak at 4,827 *m*/*z* belonging *S. indica* compared with *S. algae* and *S. chilikensis*. **(B)** Different distribution of peaks at 4,221, 3,778, and 9,574 *m*/*z* between *S. xiamenensis* and *S. seohaensis.*

Due to the misidentification of nine strains by MALDI-TOF MS, we analyzed the spectra data of the type strains to determine whether there are potential species-specific biomarker peaks that can be used to improve the accuracy. The output file of peak intensity for different type strains was generated by MDT Master Software. After the mass spectrum peaks with relative intensity < 2% were removed, the remaining peaks were collected for further analysis. Owing to the non-normal data, *P* < 0.05 (Kruskal–Wallis *H* test) was considered significant in peak distribution. Biomarker peaks were detected using peak lists among 36 type strains (Table 2), and 125 test strains were examined for verification. The potential species-specific biomarker peaks were located within the range of 2,000–12,000 *m*/*z*. The nine wrongly identified strains were correctly identified at the species level by using the biomarker peaks. An average of eight species-specific peaks was found for each type strain, while *S. aestuarii* JCM 17801^T^ harbored 21 specific peaks, higher than the average amount. Furthermore, *S. aestuarii*, *S. aquimarina*, *S. baltica*, *S. carassii*, and *S. gaetbuli* type strains harbored multiple specific peaks in a relative-low range (2,000–3,000 *m*/*z*). In the comparison of MS fingerprinting among *S. algae*–*S. chilikensis*–*S. indica*, the specific peaks were 10,065 *m*/*z* (*S. algae*), 3,307 *m*/*z* (*S. chilikensis*), and 4,827 *m*/*z* (*S. indica*) (Figure 4A). Similarly, for the *S. xiamenensis*–*S. seohaensis* group, the peak with 4,221 *m*/*z* only appeared in *S. xiamenensis*, while peaks of 3,778 *m*/*z* and 9,574 *m*/*z* were present in *S. seohaensis* (Figure 4B).

**TABLE 2:** Species-specific biomarker peaks of 36 *Shewanella* type strains.

No.	Species	Strain	Species-specific biomarker peaks
1	*Shewanella aestuarii*	JCM 17801^T^	2,784, 2,956, 2,970, 3,874, 3,882, 4,323, 4,345, 4,915, 5,209, 5,230, 5,913, 6,816, 6,827, 6,838, 7,751, 7,762, 7,769, 9,810, 9,831, 9,851, 10,459
2	*Shewanella algae*	JCM 21037^T^	4,654, 4,661, 5,086, 5,347, 6,163, 6,231, 6,534, 8,641, 10,065
3	*Shewanella algicola*	KCTC 23253^T^	2,816, 3,793, 5,309, 5,663, 8,999, 9,196, 9,452, 9,932, 11,344
4	*Shewanella algidipiscicola*	LMG 23746^T^	4,445, 4,531, 4,635, 4,834, 4,843, 5,109, 6,658, 7,309, 9,043
5	*Shewanella aquimarina*	JCM 12193^T^	3,854, 7,710, 8,582
6	*Shewanella baltica*	DSM 9439^T^	2,700, 4,061, 5,443, 6,307, 7,564, 8,409, 10,887, 10,887
7	*Shewanella basaltis*	KCTC 22121^T^	3,495, 3,602, 4,288, 4,518, 6,363, 6,656, 6,747, 11,044
8	*Shewanella carassii*	08MAS2251^T^	2,681, 2,706, 2,710, 2,859, 2,948, 4,018, 6,823, 8,952, 11,264
9	*Shewanella chilikensis*	KCTC 22540^T^	3,307, 4,034, 4,775, 4,781, 4,984, 6,028, 7,279, 7,316, 8,585, 9,525, 9,970
10	*Shewanella corallii*	DSM 21332^T^	2,290, 2,614, 2,906, 3,615, 4,849, 5,478, 7,216, 7,232, 8,742, 9,699, 10,957
11	*Shewanella decolorationis*	JCM 21555^T^	4,924, 6,357, 7,613, 8,460, 9,112, 10,652, 10,795
12	*Shewanella dokdonensis*	KCTC 22898^T^	3,644, 3,808, 4,319, 4,846, 5,064, 5,083, 5,369, 6,132, 7,619, 10,146, 10,319
13	*Shewanella electrodiphila*	DSM 24955^T^	2,238, 6,916, 7,409, 8,593, 9,156, 9,279, 9,325, 11,135
14	*Shewanella gaetbuli*	KCTC 22431^T^	2,171, 2,865, 5,285, 6,311, 6,541, 7,601
15	*Shewanella gelidii*	MCCC 1K00697^T^	4,384, 4,957, 5,547, 5,602, 5,990, 9,926, 9,941, 9,957
16	*Shewanella glacialipiscicola*	LMG 23744^T^	3,367, 4,625, 6,790, 11,193
17	*Shewanella hafniensis*	KCTC 22180^T^	3,552, 4,855, 5,495, 5,892, 7,343, 8,413
18	*Shewanella hanedai*	DSM 6066^T^	3,937, 4,858, 7,300, 7,442, 7,469, 9,715, 10,243
19	*Shewanella indica*	KCTC 23171^T^	2,693, 2,717, 2,805, 3,425, 3,461, 4,148, 4,827, 6,557, 9,066, 9,482
20	*Shewanella inventionis*	KCTC 42807^T^	5,674, 5,997, 8,172, 11,230, 11,303, 11,319
21	*Shewanella kaireitica*	DSM 17170^T^	4,113, 4,186, 4,685, 5,124, 5,191, 6,280, 6,739, 6,758, 10,225
22	*Shewanella litorisediminis*	KCTC 23961^T^	2,552, 2,713, 3,034, 3,886, 3,909, 4,496, 4,651, 5,483, 6,435, 6,626, 10,965
23	*Shewanella livingstonensis*	LMG 19866^T^	3,819, 4,805, 7,639, 9,974
24	*Shewanella mangrovi*	MCCC 1A00830^T^	2,802, 4,246, 5,453, 5,677, 10,029, 10,906, 11,371
25	*Shewanella marinintestina*	JCM 11558^T^	3,458, 4,550, 6,224, 6,336, 7,429, 7,569, 9,060, 9,263, 10,388, 11,169, 11,176
26	*Shewanella marisflavi*	JCM 12192^T^	3,527, 3,555, 5,469, 6,053, 6,204, 7,306, 9,239, 10,237, 10,937
27	*Shewanella olleyana*	LMG 21437^T^	2,174, 2,329, 6,695, 7,530, 9,057, 9,143
28	*Shewanella pacifica*	KCTC 12235^T^	3,500, 11,274
29	*Shewanella pneumatophori*	KCTC 23973^T^	2,642, 5,196, 11,019, 11,129, 11,137, 11,214, 11,224
30	*Shewanella profunda*	JCM 12080^T^	3,737, 4,104, 5,149, 5,329, 7,477, 8,956, 8,977, 10,070, 10,858
31	*Shewanella putrefaciens*	ATCC 8071^T^	3,365, 3,759, 4,509, 4,664, 6,351, 7,552, 9,659, 10,098, 10,867
32	*Shewanella sairae*	MCCC 1A01705^T^	6,546, 7,474, 8,579, 9,460, 9,640, 11,029, 11,125
33	*Shewanella schlegeliana*	JCM 11561^T^	8,254, 11,161
34	*Shewanella seohaensis*	KCTC 23556^T^	2,448, 3,172, 3,778, 4,454, 4,823, 6,129, 7,159, 8,501, 9,574
35	*Shewanella vesiculosa*	LMG 24424^T^	4,760, 4,790, 4,998, 6,652, 6,668, 9,071
36	*Shewanella xiamenensis*	MCCC 1A00763^T^	3,371, 3,610, 3,658, 4,221, 5,417, 8,443, 10,089, 10,834

## Discussion

Multiple *Shewanella* species are frequently isolated from food products and clinical specimens as opportunistic pathogens (Pagani et al., 2003; Liu et al., 2013; Janda and Abbott, 2014). Commercial systems, such as Vitek and MALDI-TOF MS (bioMérieux, Bruker), are available for species identification in clinical laboratories (Regoui et al., 2020; Sánchez-Juanes et al., 2020). However, only a few species, like *S. putrefaciens* and *S. algae*, were recorded in the database (Liu et al., 2013; Janda and Abbott, 2014; Zhang et al., 2018). Several other *Shewanella* species have often been misidentified by biochemical tests or MALDI-TOF MS (Byun et al., 2017; Zhang et al., 2018). Therefore, we urgently need an expanded database to identify *Shewanella* species correctly.

MALDI-TOF MS has revolutionized the routine identification of microorganisms in clinical laboratories by introducing a simple, rapid, high-throughput, and low-cost technology (O’Connor et al., 2016; Bao et al., 2018). The two key requirements of MALDI-TOF MS microbial identification are the species coverage in the database and the representativeness of bacterial diversity used for the database construction (Rodriguez-Temporal et al., 2017; Honnavar et al., 2018; Paul et al., 2019).

In this study, the PMRS database included the type strains of 36 *Shewanella* species for MALDI-TOF MS identification at both genus and species levels. The MSP dendrogram was used to evaluate the distance and relationship of the type strains that represent the 36 *Shewanella* species. The tree topology confirmed that each species is well separated. The previously recognized clades, i.e., Algae, Gelidii, Aquimarina, and Putrefaciens, were also grouped together respectively as expected, confirming the spectra contained sufficient signal for species identification. The identification accuracy of the MALDI-TOF MS was validated using 125 test strains, the species identity of which were confirmed by MLSA. The 125 test strains analyzed belonged to six different species, namely, *S. algae*, *S. chilikensis*, *S. indica*, *S. xiamenensis*, *S. seohaensis*, and *S. carassii.* Thus, we can confidently conclude that the MALDI-TOF MS method developed here is capable of identifying *Shewanella* species. Although there are over 70 *Shewanella* species and other species remain to be tested, these six species are the most commonly isolated species from clinical samples, and other species are relatively rare (Zhang et al., 2018). The testing of other 30 *Shewanella* species included in this database and additional species not included in this study will further expand the utility of this method.

However, in our study, nine out of 125 strains were misidentified. These nine isolates belonged to five species, which were divided into two groups, *S. algae*–*S. chilikensis*–*S. indica* and *S. xiamenensis*–*S. seohaensis*. The strains of each group shared the most recent common ancestor in the MLSA phylogenetic tree (Figure 2), suggesting a close evolutionary relationship of the species within each group. The close relatedness is likely to be the cause of the misidentification, as MALDI-TOF MS primarily uses main peaks represented by ribosomal proteins in the spectrum for strain identification (Ryzhov and Fenselau, 2001; Bremer and Dennis, 2008; Nakamura et al., 2016). It is known to be difficult to distinguish by MALDI-TOF MS *Shigella* spp. from *E. coli* (Wieser et al., 2012), *Brucella melitensis* from *Ochrobactrum anthropi* (Poonawala et al., 2018), and some species within the genus *Bacillus* (McLaughlin et al., 2014), as there is very little difference between them in the spectra of ribosomal proteins.

In order to overcome the limitations of MALDI-TOF MS in differentiating closely related species, potential species-specific biomarker peaks were found to be useful. Peaks in the range of 2,000–20,000 *m*/*z* are more likely to be ribosomal proteins that are discriminatory at the species level. Ha et al. (2019) successfully applied low-mass profiling to identify species-specific mass peaks for the identification of two genetically closely related *Bacillus* species. In this study, we took a similar approach and identified species-associated biomarker peaks based on the type strains, validated using the 125 test strains. With the use of the species biomarker peaks, nine misidentified test strains can be accurately identified at the species level. We identified 2–21 species-associated biomarkers for the species level identification. However, it should be noted that these species-associated biomarkers were identified based on one type strain; and thus when more strains of a given species are included, some of these markers may become variable within a species or non-species specific. For the species with multiple test strains available, the species-associated biomarker appears to be species specific.

## Conclusion

The establishment of the PMRS library provides the technical basis for the detection and identification of *Shewanella* species that are relevant to food safety and clinical disease. The study revealed that MALDI-TOF MS could be a fast and relatively inexpensive method for the identification of the *Shewanella* genus. Species-specific biomarker peaks were identified and employed to improve the identifications at the species level. MALDI-TOF MS can effectively replace traditional identification methods for the identification of *Shewanella*.

## Data Availability Statement

The original contributions presented in the study are included in the article/Supplementary Materials, further inquiries can be directed to the corresponding author/s.

## Author Contributions

DW designed the work. KY and ZH performed the experiments. KY, ZH, YL, QF, LL, SW, HD, HC, and YX collected the samples and isolated strains. KY, ZH, and RL wrote the manuscript. All authors contributed to the article and approved the submitted version.

## Conflict of Interest

QF, LL, and SW were employed by the company Zybio Inc. Chongqing, China. The remaining authors declare that the research was conducted in the absence of any commercial or financial relationships that could be construed as a potential conflict of interest.

## Footnotes

1 http://www.bacterio.net/shewanella.html
