# Supplementary material for: Establishment and Application of Matrix-Assisted Laser Desorption/Ionization Time-of-Flight Mass Spectrometry for Detection of Shewanella Genus
Source: Front Microbiol. 2021 Feb 18;12:625821. doi: 10.3389/fmicb.2021.625821 (PMC7930330; doi:10.3389/fmicb.2021.625821)
Supplement: Supplementary file 1 [file Table_1.docx]

Table S1. GenBank numbers of housekeeping genes of the 36 *Shewanella* type strains.

| No. | Species | Strain | *gyrA* | *gyrB* | *infB* | *recN* | *rpoA* | *topA* |
| --- | --- | --- | --- | --- | --- | --- | --- | --- |
| 1 | *S. aestuarii* | JCM 17801^T^ | MH090144 | KC175545 | MH090203 | MH090245 | MH090287 | MH090329 |
| 2 | *S. algae* | JCM 21037^T^ | MH090145 | AF005686 | MH090204 | MH090246 | MH090288 | MH090330 |
| 3 | *S. algicola* | KCTC 23253^T^ | MH090146 | KP748519 | MH090205 | MH090247 | MH090289 | MH090331 |
| 4 | *S. algidipiscicola* | LMG 23746^T^ | MH090147 | MH090186 | MH090206 | MH090248 | MH090290 | MH090332 |
| 5 | *S. aquimarina* | JCM 12193^T^ | MH090148 | FJ589042 | MH090207 | MH090249 | MH090291 | MH090333 |
| 6 | *S. baltica* | DSM 9439^T^ | MH090150 | AB231331 | MH090209 | MH090251 | MH090293 | MH090335 |
| 7 | *S. basaltis* | KCTC 22121^T^ | MH090151 | FJ589041 | MH090210 | MH090252 | MH090294 | MH090336 |
| 8 | *S. carassii* | 08MAS2251^T^ | MH090153 | MF164484 | MH090212 | MH090254 | MH090296 | MH090338 |
| 9 | *S. chilikensis* | KCTC 22540^T^ | MH090154 | HM016091 | MH090213 | MH090255 | MH090297 | MH090339 |
| 10 | *S. corallii* | *DSM 21332*^T^ | MH090155 | MH090189 | MH090214 | MH090256 | MH090298 | MH090340 |
| 11 | *S. decolorationis* | JCM 21555^T^ | MH090156 | MH090190 | MH090215 | MH090257 | MH090299 | MH090341 |
| 12 | *S. dokdonensis* | KCTC 22898^T^ | MH090157 | GQ132057 | MH090216 | MH090258 | MH090300 | MH090342 |
| 13 | *S. electrodiphila* | DSM 24955^T^ | MH090158 | MH090191 | MH090217 | MH090259 | MH090301 | MH090343 |
| 14 | *S. gaetbuli* | KCTC 22431^T^ | MH090160 | KC175546 | MH090219 | MH090261 | MH090303 | MH090345 |
| 15 | *S. gelidii* | MCCC 1K00697^T^ | MH090161 | KT899956 | MH090220 | MH090262 | MH090304 | MH090346 |
| 16 | *S. glacialipiscicola* | LMG 23744^T^ | MH090162 | MH090193 | MH090221 | MH090263 | MH090305 | MH090347 |
| 17 | *S. hafniensis* | KCTC 22180^T^ | MH090163 | MH090194 | MH090222 | MH090264 | MH090306 | MH090348 |
| 18 | *S. hanedai* | DSM 6066^T^ | MH090165 | AF005693 | MH090224 | MH090266 | MH090308 | MH090350 |
| 19 | *S. indica* | KCTC 23171^T^ | MH090166 | HM016092 | MH090225 | MH090267 | MH090309 | MH090351 |
| 20 | *S. inventionis* | KCTC 42807^T^ | MH090168 | MH090196 | MH090227 | MH090269 | MH090311 | MH090353 |
| 21 | *S. kaireitica* | DSM 17170^T^ | MH090169 | MH090197 | MH090228 | MH090270 | MH090312 | MH090354 |
| 22 | *S. litorisediminis* | KCTC 23961^T^ | MH090170 | JQ824140 | MH090229 | MH090271 | MH090313 | MH090355 |
| 23 | *S. livingstonensis* | LMG 19866^T^ | MH090171 | MH090198 | MH090230 | MH090272 | MH090314 | MH090356 |
| 24 | *S. mangrovi* | MCCC 1A00830^T^ | MH090172 | JPEO01000023 | MH090231 | MH090273 | MH090315 | MH090357 |
| 25 | *S. marinintestina* | JCM 11558^T^ | MH090173 | AB081763 | MH090232 | MH090274 | MH090316 | MH090358 |
| 26 | *S. marisflavi* | JCM 12192^T^ | MH090174 | FJ589037 | MH090233 | MH090275 | MH090317 | MH090359 |
| 27 | *S. olleyana* | LMG 21437^T^ | MH090175 | MH090199 | MH090234 | MH090276 | MH090318 | MH090360 |
| 28 | *S. pacifica* | KCTC 12235^T^ | MH090176 | MH090200 | MH090235 | MH090277 | MH090319 | MH090361 |
| 29 | *S. pneumatophori* | KCTC 23973^T^ | MH090177 | MH090201 | MH090236 | MH090278 | MH090320 | MH090362 |
| 30 | *S. profunda* | JCM 12080^T^ | MH090178 | FJ589036 | MH090237 | MH090279 | MH090321 | MH090363 |
| 31 | *S. putrefaciens* | ATCC 8071^T^ | MH090179 | AF005669 | MH090238 | MH090280 | MH090322 | MH090364 |
| 32 | *S. sairae* | MCCC 1A01705^T^ | MH090180 | AB081768 | MH090239 | MH090281 | MH090323 | MH090365 |
| 33 | *S. schlegeliana* | JCM 11561^T^ | MH090181 | AB081766 | MH090240 | MH090282 | MH090324 | MH090366 |
| 34 | *S. seohaensis* | KCTC 23556^T^ | MH090182 | GU944673 | MH090241 | MH090283 | MH090325 | MH090367 |
| 35 | *S. vesiculosa* | LMG 24424^T^ | MH090184 | MH090202 | MH090243 | MH090285 | MH090327 | MH090369 |
| 36 | *S. xiamenensis* | MCCC 1A00763^T^ | MH090185 | FJ589040 | MH090244 | MH090286 | MH090328 | MH090370 |
